# The effect of colloidal solution of molybdenum nanoparticles on the microbial composition in rhizosphere of *Cicer arietinum* L.

**DOI:** 10.1186/1556-276X-9-289

**Published:** 2014-06-09

**Authors:** Natalia Yu Taran, Olena M Gonchar, Kostyantyn G Lopatko, Lyudmila M Batsmanova, Mykola V Patyka, Mykola V Volkogon

**Affiliations:** 1 Institute of Biology, Taras Shevchenko National University of Kyiv, 64/13, Volodymyrska Street, Kyiv 01601, Ukraine; 2 National University of Life and Environmental Sciences of Ukraine, 12 Heroiv Oborony, Kyiv 03041, Ukraine

**Keywords:** Nanoparticles, Colloidal solution, Molybdenum, Chickpea, Microorganisms, Catalase, Symbiosis

## Abstract

The use of colloidal solutions of metals as micronutrients enhances plant resistance to unfavorable environmental conditions and ensures high yields of food crops due to the active penetration of nanoelements into the plant cells.

Microbiological examination of rhizosphere soil have revealed that combined use of colloidal solution of nanoparticles of molybdenum (CSNM, 8 mg/l), and microbial preparation for pre-sowing inoculation of chickpea seeds stimulates the development of ‘agronomically valuable’ microflora. It was shown that combined seed treatment with colloidal solution of Mo nanoparticles with microbial preparation have stimulated nodule formation per plant by four times compared to controls. Single treatment with CSNM increased the number of nodules by two times, while the treatment of microbial preparation have not significantly affected the number of nodules per plant.

**PACS:**

Colloids, 82.70.Dd; Ecology, 87.23.-n

## Background

Creation of materials easily assimilated by living creatures and not harmful to the environment is one of the important issues of modern nanotechnologies. These are the requirements that can ensure materials functionality as nanobiomaterials. For the last years, lots of experiments were performed in order to define the effect of nanobiomaterials on crop production [1,2]. Thus, it is known that nanoparticles have positive morphological effects like enhancement of seed germination rates, improvement of root and shoot formation and their ratio, as well as accumulation of vegetative biomass of seedlings in many crop plants [3]. Nanoparticles influence on the cell level thus increasing the pace of physiological processes in plants. Nanoparticles of zinc, cuprum, iron, etc., received by now are up to 40 times less toxic than the salts [4,5]. They are gradually absorbed while their ionic forms are immediately included into the biochemical reactions. By taking part in electron transfer, nanoparticles increase the activity of plant enzymes, promote conversion of nitrates to ammonia, intensify plant respiration and photosynthesis processes, synthesize enzymes and amino acids, and enhance carbon and nitrogen nutrition and thus have a direct influence on the plant mineral nutrition [6-8]. Chickpea, an annual plant of the legume family, is widespread in countries with subtropical and tropical climates - India, Pakistan, Turkey, Iran, Australia, etc. Among the legumes, chickpeas are characterized by high nutritional value, amount of vitamins, and other biologically valuable substances which in turn causes high demand for this grain crop used for food and feed purposes [9]. Resistance to high temperatures and global climate changes have created the favorable conditions for the formation of high yields of chickpea and attract the attention of producers of agricultural products. Chickpea plants are drought tolerant and are able to fix atmospheric nitrogen by forming the symbiotic relationships with nitrogen fixation microorganisms that not only meet the requirements of plants in nitrogen but also bring it into the ground [10].

Most biotechnologies developed for the southern regions do not give the desired effect in other climatic zones [5,10]. The colloidal solutions containing biologically active metals are now being widely used along with traditional biological preparations. There are preliminary conclusions about the positive effects of these preparations on the productivity and plant resistance to adverse environmental factors [11]. This is especially important for growing plants on problem soils, i.e., soils which have vital mineral elements in inaccessible to plant forms that lead to inhibition of plant growth and decrease of yields [1,10].

The level of productivity of crops is largely determined by the soil microbial communities and their function [12]. Processes specific to each group of soil microbiota are complicated and usually are closely related to the population activity of bacteria. Reported toxic effects of nanoparticles even more determine the necessity of the comprehensive research of colloidal solutions of metals prior to their use in agriculture.

Taking this into account, we considered that an important step is to compare the impact of the traditional techniques of biotechnology (microbial preparation) and application of colloidal solution of metals, as well as the complex use of conventional and nanotechnology on the composition of microbiota of the plant rhizosphere.

## Methods

*Colloidal solution of molybdenum nanoparticles* was obtained by means of metal dispersion by electric current pulses with an amplitude of 100 to 2,000 A in water [13]. With this, the master colloidal solution contains an aqueous solution of molybdenum nanoparticles with concentration of not less than 8 mg/l. The size of nanoparticles of metals is from 100 to 250 nm and their concentration in bidistilled water is not more than the value calculated by formula 1.

(1) m≤1278×V−0.8

where *m* is the concentration of nanoparticles of metal (mg/l) and *V* is the volume of 1 mole of metal atoms (cm^3^/mol).

The colloidal solution of nanoparticles of molybdenum was used in the dose of 1 microliter per gram (μl/g).

*Microbial preparation* used in our experiments is registered in Ukraine trademark and is included into the list of pesticides and agrochemicals permitted for use in Ukraine. As an active agent, the highly competitive strains of *Bradyrhizobium japonicum* were used, adapted to the soil and climatic conditions of Ukraine. The concentration of bacteria in 1 g of preparation is not less than 6 × 10^8^ cells. The preparation was used in accordance to the manufacturer's instructions in a dose of 200 g per 1.2 l of water per hectare of seed rate that corresponds to 10^6^ of bacteria cell concentration per single seed.

*Experiments* were performed in stationary conditions at the Agronomy Department of Plant Experimental Station of National University of Life and Environmental Sciences of Ukraine on typical gray, light sandy loam soils. Chickpea seed inoculation was carried out for 1 to 2 h before sowing. The seeds were dampened with water (2% by weight) in control variant, aqueous suspension of microbial preparation, colloidal solution of molybdenum nanoparticles alone and in combination with microbial preparation.

The scheme of the experiment is as follows:

1. Control (water treatment)

2. Colloidal solution of nanoparticles of molybdenum (CSMN)

3. Microbial preparation

4. Microbial preparation + CSMN

*Determination and quantification of basic physiological groups of microorganisms* in rhizosphere soil of chickpea plants was performed using standard microbiological methods [14]. Sowing of microorganisms on culture media are made of 10^−3^ dilutions (fungi and cellulose destructive bacteria) and 10^−4^ (other microorganisms). Sowing of each dilution was performed at least three times. Calculation of the total number of microorganisms on nutrient media was performed on the third, fifth, and seventh day of incubation. After counting the number of colonies on the surface, the number of microorganisms in 1 ml of the appropriate dilution was determined. In the estimation of the number of cells of microorganisms in 1 g of wet soil, the result obtained was multiplied by the degree of dilution (10^3^, 10^4^, 10^5^, etc.). To determine the number of microorganisms in 1 g of dry soil, the respective number of cells in 1 g of wet soil was multiplied by a correction factor of soil moisture [12,14].

*Direction of microbiological processes* in rhizosphere soil of chickpea plants was determined using the coefficient of mineralization, which allows to characterize the processes and intensiveness of mineralization processes and oligotrophic index that characterizes the oligotrophic degree of microbial communities. The direction and intensity of individual microbial transformation processes of nitrogen was estimated by the ratio of the number of microorganisms of respective ecological trophic groups, which were determined by cultivation of soil suspensions on solid culture media In the index of mineralization, immobilization was calculated by the ratio of the number of microorganisms that metabolize mineral and organic nitrogen (KAA/MPA); the oligotrophic rate is the ratio of oligotrophic microorganisms and the total number of microorganisms on the MPA and KAA media. The rate of microbial transformation of organic matter of the soil was calculated by the total number of microorganisms on the MPA and KAA and mineralization rate [12,14].

*Formation of symbiotic systems* was determined by calculating the weight and number of nodules formed on roots of chickpea plants.

*Formation of plant resistance to phytopathogens* was determined by the activity of oxidoreductase enzyme catalase using the spectrophotometric method by Aeby [15]. In this method, the 250 mg of plant tissue was comminuted in frozen mortar with 0.5 extraction buffer (50 mM K, Na-phosphate buffer, рН 7.8). Homogenate was centrifuged for 5 min at 12,000 g and placed into the refrigerator (4°C). Then, 30 μl of plant extract was added to 2.95 ml of 50 mМ K,Na-phosphate buffer (рН 7.0). The reaction was initiated by adding 20 μl of 0.6 M hydrogen peroxide to the reaction mixture. Determination of decay rate of hydrogen peroxide by catalase in studied sample was determined by measuring the changes of absorbency of the mixture at 240-nm wavelength for each second within the 100-s time frame.

Calculations of catalase activity in corresponding units per 1 mg of protein [16] in the following formula (2) was used:

(2) $$A=\left(\left(\mathrm{\Delta D}/T\right)Х\right)/\left(L\times С\right)$$

where *A* is the enzyme activity; Δ*D* is the absorbency fluctuation; *X* is the final dilution of plant extract in cuvette; *T* is the reaction time, s; *L* is the layer width, mm; and *С* is the protein content in sample, mg.

*Statistical analysis* of the results was performed using the software package Sigma Stat - 6.0 and Microsoft Excel 2010.

## Results and discussion

*The dynamics of soil microorganism development* under the influence of molybdenum nanoparticles along and in combination with microbial preparation are presented in Tables 1 and 2. The number of nitrifying microorganisms in the variants with CSNM at crop-emerging stage was higher than in control variants by 75.2%, while the joint application of CSNM and microbial preparation had almost doubled that number. At flowering stage, the number of nitrifying microorganisms in the variants with CSMN had grown by 115%, while that in the variants of combined use, by 35%.

**Table 1:** Development of soil microorganisms of various ecological and functional groups at plant emerging stage

Variant*	**Number of microorganisms, millions of CFU/1 g of dry soil**									
	**Nitrifiers**	**Spore forming**	**Oligotrophs**	**Ammonifier**	**Pedotrophs**	**Actynometes**	**Microorganisms that utilize mineral forms of nitrogen**	**Azotobacter**	**Phosphorousmobilizing**	**Cellulose destructive**
No. 1	25.23 ± 1.26	4.59 ± 0.23	32.88 ± 1.64	19.12 ± 0.96	10.71 ± 0.54	3.06 ± 0.15	31.35 ± 1.57	5.35 ± 0.27	16.06 ± 0.80	9.18 ± 0.46
No. 2	43.82 ± 2.19	14.85 ± 0.74	63.87 ± 3.19	11.14 ± 0.56	14.85 ± 0.74	7.43 ± 0.37	65.35 ± 3.27	4.46 ± 0.22	36.39 ± 1.82	11.14 ± 0.56
No. 3	22.64 ± 1.13	7.20 ± 0.36	54.88 ± 2.74	22.64 ± 1.13	17.15 ± 0.86	2.06 ± 0.10	65.17 ± 3.26	4.12 ± 0.21	34.30 ± 1.72	13.03 ± 0.65
No. 4	57.10 ± 2.86	16.53 ± 0.83	15.03 ± 0.75	38.32 ± 1.92	6.01 ± 0.30	11.27 ± 0.56	62.36 ± 3.12	7.51 ± 0.38	31.56 ± 1.58	12.77 ± 0.64

These are taken in the root zone of chickpea plants *Cicer arietinum* L. at pre-sowing seed treatment with colloidal solution of nanoparticles of molybdenum, microbial preparation, and their combination *1 - Control (water treatment), 2 - colloidal solution of nanoparticles of molybdenum (CSMN), 3 - microbial preparation, 4 - microbial preparation + CSMN.

**Table 2:** Development of soil microorganisms of various ecological and functional groups at plant flowering stage

Variant*	**Number of microorganisms, millions of CFU/1 g of dry soil**									
	**Nitrifiers**	**Spore forming**	**Oligotrophs**	**Ammonifier**	**Pedotrophs**	**Actynometes**	**Microorganisms that utilize mineral forms of nitrogen**	**Azotobacter**	**Phosphorous mobilizing**	**Cellulose destructive**
No. 1	6.68 ± 0.33	8.91 ± 0.45	5.94 ± 0.30	8.91 ± 0.45	3.71 ± 0.19	3.71 ± 0.19	1.49 ± 0.07	0	0	14.85 ± 0.74
No. 2	14.41 ± 0.72	4.12 ± 0.21	25.38 ± 1.27	8.23 ± 0.41	66.54 ± 3.33	5.49 ± 0.27	9.60 ± 0.48	6.86 ± 0.34	0	39.79 ± 1.99
No. 3	24.47 ± 1.22	0.76 ± 0.04	15.29 ± 0.76	19.12 ± 0.96	33.65 ± 1.68	8.41 ± 0.42	3.06 ± 0.15	1.53 ± 0.08	4.59 ± 0.23	52.00 ± 2.60
No. 4	9.02 ± 0.45	0.75 ± 0.04	23.29 ± 1.16	8.26 ± 0.41	122.47 ± 6.12	6.01 ± 0.30	11.27 ± 0.56	6.01 ± 0.30	2.25 ± 0.11	19.53 ± 0.98

These are taken in the root zone of chickpea plants *Cicer arietinum* L. at pre-sowing seed treatment with colloidal solution of nanoparticles of molybdenum, microbial preparation, and their combination.*1 - Control (water treatment), 2 - colloidal solution of nanoparticles of molybdenum (CSMN), 3 - microbial preparation, 4 - microbial preparation + CSMN.

The pre-sowing seed treatment of chickpea plants with colloidal solution of nanoparticles of molybdenum had promoted the development of oligotrophic bacteria in the rhizosphere which exceeded the control value by 94% at plant emerging and by 3.2 times - at flowering stage. Concomitant use of CSNM with microbial preparation also had the positive influence on the number of oligotrophs during the flowering stage increasing their number by 2.9 times in comparison to the control variant. However, bacteria count during the plant emerging stage had showed the decrease of a number of oligotrophic microorganisms by 54% comparing to control.

In the use of nanobiotechnologies, the number of phosphorous-mobilizing bacteria during the emerging stages was two times higher than that in the control, while that in the combined use of CSNM with microbial preparation also increased. During the flowering stage, the number of phosphorous-mobilizing microorganisms was negligible. Thus, they were not determined in the control variant and in plants treated with the CSNM but only in variants with microbial preparation - their number was between 2.25 and 4.58 million CFU per 1 g of dry soil.

The study of changes in the number of microorganisms that break down cellulose in variants with CSNM application had revealed the increase number of bacteria and fungi by 21%. The combined use of CSNM and microbial preparation had promoted 39% increase of this number as compared to the control during the emerging stage. During flowering stage, the number of cellulose-destructive microorganisms had steadily increased in the variants with nanoparticle treatment. Thus, the number of cellulose-destructive bacteria in soil of plant treated with CSNM was 1.6 times greater than that in the control, while that at joint use with microbial preparation, by 31.5%.

The total number of ammonifiers in the variants with CSMN was higher only by 0.5%, while that in the combined treatment had doubled their number in comparison with that in the control. During the flowering stage, no significant changes in the quantity of microorganisms of this group were observed. Quantification of pedotrophic bacteria also indicates the growth of microorganisms of these groups.

The 2 to 2.5-time increase of the number of microorganisms that utilize mineral forms of nitrogen was observed in variants with CSNM during the whole vegetation period.

The number of actinomycetes in variants with application of CSNM was 1.4 to 2.7 times higher than in controls. During the flowering stage, these figures had exceeded the control by 48% to 61%.

The number of spore-forming microorganisms had varied between the plant developmental stages. Thus, at the emerging stage in variants with CSNM application, the number of spore-forming microorganisms was higher, 2.2 to 2.6 times, while the opposite numbers were obtained during the flowering stage - the quantity of spore-forming microorganisms was reduced by 53% to 91% compared to that of the control.

The number of microscopic fungi in variants with CSNM at the beginning of the growing season (emerging stage) had exceeded the control value by 84%, and during the flowering stage - 3.1 times. Joint use of colloidal solution of nanoparticles of molybdenum with microbial preparation had also a positive effect on the number of micromycetes. Thus, this number had increased by 20% during the emerging stage and by 52.9% at the flowering stage compared to that of control.

The performed comprehensive microbiological examination of soil had showed that pre-sowing inoculation of chickpea seeds with CSNM at a dose of 8 mg/l in combination with microbial preparation was the optimal system for enhancement of the plant root nutrition by the optimization of microbiological fertilization of plants and effect of colloidal solution of nanoparticles of molybdenum through the promotion of development of active ‘agronomically valuable’ microflora.

### Direction of microbiological processes

**Figure 1:**
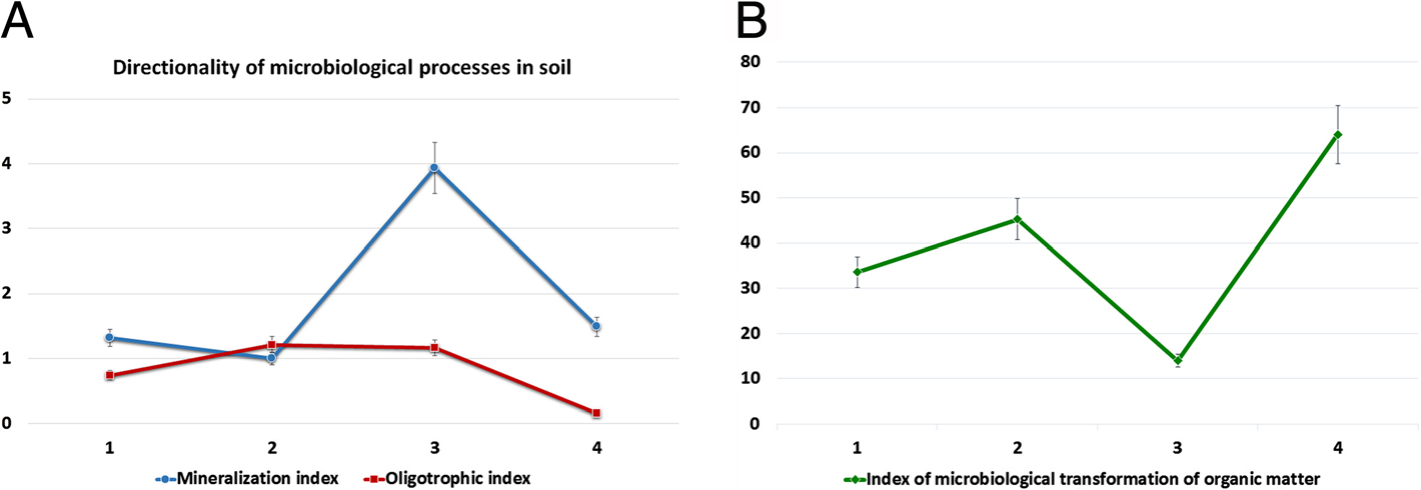
**Performance orientation of microbial processes in rhizosphere soil of chickpea plants.** Plant emerging stage: (1) Control (water treatment), (2) colloidal solution of nanoparticles of molybdenum (CSMN), (3) microbial preparation, (4) microbial preparation + CSMN.

**Figure 2:**
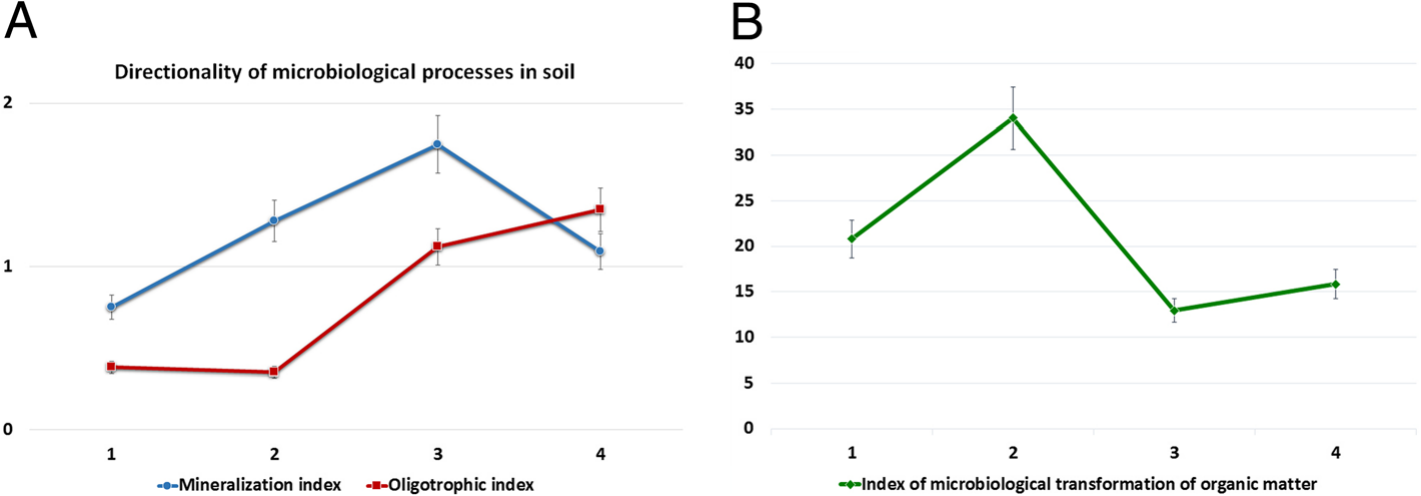
**Performance orientation of microbial processes in rhizosphere soil of chickpea plants.** Plant flowering stage: (1) Control (water treatment), (2) colloidal solution of nanoparticles of molybdenum (CSMN), (3) microbial preparation, (4) microbial preparation + CSMN.

The study of microbiological processes in the soil allows deeper analysis of changes in the structure of soil and biotic system. The focus of microbiological processes was determined using the mineralization coefficient, which permits to characterize the intensity of mineralization processes and oligotrophic index of microbial communities.It was noted that the intensity of mineralization processes was higher in variants with colloidal solution of nanoparticles of molybdenum. It should be noted that this tendency was observed in both variants with CSNM application (3.93 to 1.94). The intensity had decreased in the flowering stage, but still the figure in experimental variants was higher than in the control (1.75 to 1.35) (Figure 1).The oligotrophic index of soils in variants with application of CSNM and microbial preparation was lowest (0.16) indicating the optimal conditions for the formation of soil microcoenosis. At this, the significant increase of number of oligotrophic microorganisms developed due to the minimal amount of organic matter in the soil and typical for the last stages of mineralization is of big interest. Thus, the oligotrophic index of soil during the flowering stage was two times higher and reached 1.35 (Figure 2). Doubling of oligotrophic index had reflected the changes in the structure of soil microbial coenosis.

The application of colloidal solution of nanoparticles of molybdenum had enhanced the development of almost all groups of microorganisms two to three times relative to the control, mainly due to bacteria that metabolize mineral nitrogen, associative nitrogen fixation and associative oligotrophic microorganisms, that was also confirmed by the mineralization and oligotrophic indices.

The application of CSNM in combination with bacterial preparation had a positive effect on the rate of transformation of organic matter, which increased threefold compared to that of the control, followed by the enhancement of mineralization processes and oligotrophic rates, indicating the improvement of trophic regime of the soil. These patterns can be considered as valid in all investigated soil samples.

### Formation of symbiotic systems

Plants of the legume family are able to form symbiotic systems with nitrogen-fixing rhizosphere microorganisms. Formation of legume-rhizobial symbiosis includes a number of successive stages from adsorption of bacterial cells on the surface of root hairs and infection to the formation of special symbiotic forms, bacteroides, where the complex enzyme complex, nitrogenase, is synthesized. It catalyzes the reduction of molecular nitrogen from the atmosphere [11]. This complex consists of two enzymes: the actual nitrogenase (so-called MoFe protein or dinitrogenase) and dehydrogenase (Fe protein) [17]. The MoFe protein cofactor consists of two atoms of molybdenum, which determines the relevance of a given study of influence of colloidal solution of nanoparticles of molybdenum on nodulation - central link of legume - and rhizobial symbiosis, providing the necessary conditions for the formation and functioning of the enzyme complex and nitrogen-fixing system [11,18].

The most favorable conditions for rhizobia were observed in the rhizosphere of plants treated with CSNM in combination with microbial preparation. Joint application of these preparation for pre-sowing seed treatment had increased nodule formation per plant more than four times higher than in the control variant. Single use of CSNM had allowed the increase of number and mass of nodules two times while the seed treatment with microbial preparation had not significantly affected the number of nodules per plant (Table 3). It should be noted that most of plants in the control variant had not developed root nodules.

**Table 3:** Number and mass of nodules formed on the roots of chickpea plans

**Variants**	**Number of nodules, pcs./plant**	**Mass of nodules, mg/plant**
Control (water treatment)	0.6 ± 0.03	90 ± 0.45
Colloidal solution of nanoparticles of molybdenum	6.7 ± 0.033	560 ± 2.8
Microbial preparation	3.3 ± 0.0165	770 ± 3.85
Microbial preparation + CSMN	12.8 ± 0.064	780 ± 3.9

### Plant resistance to pathogens

Plant resistance to pathogens depends on many factors, including the formation of reactive oxygen species (ROS), which is one of the least specific reactions of living organisms. ROS can promote eradication of plant pathogens by oxidative explosion and as a result of hypersensitivity reaction, there is formation of a zone of dead plant cells rich in antimicrobial compounds around the infection area. Regulation and generation of ROS is controlled by the oxidoreductase enzymes. Catalase is one of the key antioxidant enzymes of plants [19].

In our study of catalase activity of chickpea plants, it was found that colloidal solution of nanoparticles of molybdenum in a wide range of concentrations increases the activity of this enzyme more than two times compared to that of the control (Table 4) that indicates the prospects of using this solution as a promising inducer of antioxidant activity of chickpea plants, which can enhance their resistance to plant pathogens.

**Table 4:** Effect of colloidal solution of nanoparticles of molybdenum on catalase activity of chickpea plants

**Variant**	**Concentration, М**	**Catalase activity, μmol/mg protein**
Control		0.47 ± 0.0235
Colloidal solution of nanoparticles of molybdenum	10^−4^	0.91 ± 0.0455
	10^−6^	1.23 ± 0.25
	10^−8^	1.23 ± 0.25
	10^−10^	0.92 ± 0.046

## Conclusions

The proposed method of regulating plant nodulation chickpea *Cicer arietinum* L. enhances the formation of ‘agronomically valuable’ microflora and promotes positive changes in the orientation of microbiological processes in the soil, stimulation of symbiotic systems formation, and increase of antioxidant protection of chickpea plants as a result of the pre-sowing seed treatment with complex colloidal solution of nanoparticles of molybdenum and microbial preparation. The given approach is unique not only in Ukraine, but also globally in the practice of nanoparticle application in agriculture, not just in the cultivation of chickpea plants.

## Competing interests

The authors declare that they have no competing interests.

## Authors' contributions

NT performed the experimental data analysis and worked on the manuscript discussion session. OG carried out the field experimental data acquisition, quantification of basic physiological groups of microorganisms, and data analysis. KL obtained the colloidal solution of molybdenum nanoparticles. LB and MP performed the study of plants resistance formation to phytopathogens and data analysis. MV helped with the identification of microbiological processes directions and manuscript preparation, performed statistical analysis and interpretation of data. All authors read and approved the final manuscript.
